# Anesthetic management of a patient with Stiff-Person Syndrome and endometrial cancer for robotic surgery: A case report

**Published:** 2020-07-31

**Authors:** Cindy B Yeoh, Kathleen J Lee, Luis E Tollinche

**Affiliations:** 1 Memorial Sloan Kettering Cancer Center, 1275 York Avenue, New York, NY 10065; 2 Roswell Park Comprehensive Cancer Center, Elm and Carlton Streets, Buffalo, NY 14263

**Keywords:** stiff-person syndrome, cancer, anti-gad antibodies, paraneoplastic antibodies, robotic surgery

## Abstract

Stiff-person syndrome (SPS) is a rare disorder of the nervous system, characterized by muscle stiffness, rigidity, and painful spasms involving truncal and limb musculature that may severely limit mobility.

Our case documents a 53-year-old patient with SPS and endometrial cancer who was positive for anti-GAD and paraneoplastic antibodies, who presented to our institution for robotic surgery. These patients are at high risk for prolonged hypotonia and mechanical ventilation. Our patient underwent general anesthesia without complications despite multiple comorbidities.

## Introduction

Stiff-person syndrome (SPS) is a rare disorder of the nervous system, characterized by muscle stiffness, rigidity, and painful spasms involving an individual’s truncal and limb musculature to the extent of severely limiting mobility. Spasms can be triggered by sudden movements, emotional stress, and peripheral and auditory stimulation. Symptoms can progress and lead to abnormal spinal posture such as severe hunching of the spine or exaggerated lumbar lordosis. The syndrome is caused by increased muscle activity due to decreased inhibition of the central nervous system. Women are more commonly affected than men, and primary treatment consists of benzodiazepines, baclofen, and immunosuppression [1, 2].

## Case

A 53-year-old female with past medical history significant for stiff-person syndrome (SPS), non-functioning pituitary microadenoma, chronic urticaria, IgA deficiency, antinuclear (ANA) and who was paraneoplastic antibody positive, was diagnosed with endometrial cancer and presented for robotic total abdominal hysterectomy, bilateral salpingo-oophorectomy and sentinel lymph node dissection.

She had a four-year history of neurological symptoms due to autoimmune processes and spinal compression from degenerative changes. She described numbness in her hands and lower extremities, weakness limited to her lower extremities, sensation of abdominal tightness, and occasional urinary and fecal incontinence.

At the time of presentation, she had been followed by neuroimmunology for a year since her diagnosis of stiff-person syndrome. She was anti-glutamic acid decarboxylase (GAD) antibody positive and she reported symptoms as having progressed in the previous year. However, symptoms were mild and pre-operative medications only included oxycodone and tramadol. GAD autoantibody is rare and tends to be found in patients with neurological syndromes. It works against the GAD enzyme, involved in the formation of gamma aminobutyric acid (GABA). [3]

Surgery was performed with general endotracheal anesthesia. On induction, 1mg of midazolam, 60mg of lidocaine, 50mcg of fentanyl, 150mg of propofol and 50mg of rocuronium were administered. After the airway was secured, the patient was maintained on sevoflurane and a dexmedetomidine infusion was started at 0.4mcg/kg/hr for the duration of the case. The patient was placed in lithotomy, and care was taken not to excessively raise the patient’s legs in this position so as not to exacerbate lower extremity symptoms postoperatively. An additional 20mg of rocuronium was administered during the case while monitoring train of four (TOF), and the patient was reversed with 200mg of sugammadex at the conclusion of surgery. The patient emerged from anesthesia and had an uneventful recovery in the post-anesthesia care unit. She was monitored overnight and discharged home uneventfully without worsening of any preoperative SPS symptoms the next day.

## Discussion

Endometrial cancer is the most common gynecologic malignancy in the U.S. affecting 27 per 100,000 women per year [4]. A typical presentation is a post-menopausal woman with abnormal uterine bleeding who is subsequently diagnosed with endometrial cancer and treated with surgery when appropriate.

Autoimmune diseases associated with SPS are type 1 diabetes mellitus (DM1), thyroiditis, vitiligo, pernicious anemia, systemic lupus erythematosus (SLE).[2, 5] Autoimmune diseases associated with IgA deficiency may overlap with disease such as SPS with DM1, SLE, thyroiditis, autoimmune hemolytic anemia, celiac disease, and autoimmune rheumatologic diseases, to name a few. [6, 7]

Selective IgA deficiency is the most common primary autoimmune deficiency in Western world. Its etiology and pathogenesis are yet unknown due to its varied nature, but it is currently believed to be due to defect in intrinsic B cell lymphocyte maturation along with T cell abnormalities and impairment in cytokine networks.[7] This deficiency is most commonly associated with recurrent infections, allergic reactions, and autoimmune diseases and sporadically with malignancies.[6]

SPS is a rare disorder affecting 1 to 2 persons per million person per year. Pathogenesis of SPS is also yet to be fully elucidated but autoimmunity with anti-GAD antibodies or other unknown antibodies [8] or processes interfering with GABA transmission could play an important role in the clinical syndrome. Autoantibodies to GAD is seen in 60–80% of patients with SPS, but majority of SPS patients with cancers do not have autoantibodies to GAD. SPS can be seen as a paraneoplastic syndrome and is often seen with anti-amphiphysin antibodies along with anti-gephyrin antibodies and anti-Ri antibodies [1] although there have been rare reports with positive anti-GAD antibodies as paraneoplastic process. Previous cases associating cancers and positive anti-GAD antibodies in SPS patients include renal cell cancer, breast cancer, thymoma and hematologic cancers. [9]

While our patient did not have the associated malignancies typically reported with SPS or IgA deficiency, she was positive for anti-GAD antibody and paraneoplastic antibody. She had a mild, but progressively worsening SPS which could have been related to her diagnosis of endometrial cancer. To date, ours is the first report of an SPS patient with endometrial cancer and IgA deficiency who was also positive for anti-GAD and paraneoplastic antibodies.

Our patient underwent a routine anesthetic without complications despite multiple comorbidities. Several early case reports describe prolonged hypotonia/muscular weakness or prolonged mechanical ventilation either due to volatile anesthetics or neuromuscular blocking agents in patients with SPS. [10–12] since then, many more reports have demonstrated safe use of various anesthetic agents and techniques in SPS patients. Use of volatile anesthetics, muscle relaxants, regional anesthetics, and total intravenous anesthesia (TIVA) with propofol have all been described in the literature for SPS patients without complication [13–18]. Our patient received sevoflurane, rocuronium, and sugammadex along with judicious amounts of benzodiazepine, opioid and dexmedetomidine without postoperative complications. Literature supports use of Dexmedetomidine to mitigate pediatric emergence delirium. [19–22] other recent studies support the use of dexmedetomidine for decreased agitation and smoother emergence from anesthesia for adults having various types of surgeries [23–25]. Similarly, we utilized a dexmedetomidine infusion for a smoother emergence to decrease SPS triggers such as stress and stimulation associated with emergence and extubation. Minimal access robotic surgery in patients with SPS could also contribute to better postoperative courses in SPS patients because of decreased pain, faster recovery, and shorter hospital stays.

## Conclusion

Our report is the first to document positive anti-GAD antibodies in a patient with SPS, paraneoplastic process, endometrial cancer and IgA deficiency. We are also the first to report robotic surgical resection for a patient with SPS. While there are established reports of successful anesthetic management of patients with SPS with various anesthetic agents and techniques, this is also the first report describing the use of dexmedetomidine infusion for this patient population.
